# Urine Proteomic Signatures of Mild Hypothermia Treatment in Cerebral Ischemia–Reperfusion Injury in Rats

**DOI:** 10.1007/s10571-024-01483-4

**Published:** 2024-06-05

**Authors:** Dandan Zhang, Dapeng Li, Xueting Wang, Yanyan Sui, Fuguo Ma, Yuting Dai, Mingshan Wang, Weiwei Qin

**Affiliations:** 1 Department of Anesthesiology, Qingdao Municipal Hospital, Qingdao University, Qingdao, 266071 China; 2 Department of Anesthesiology, Qingdao Hospital, University of Health and Rehabilitation Sciences (Qingdao Municipal Hospital), Qingdao, 266071 China; 3 Department of Bone and Joint Surgery, Qingdao Hospital, University of Health and Rehabilitation Sciences (Qingdao Municipal Hospital), Qingdao, 266071 China

**Keywords:** Mild hypothermia, Cerebral ischemia–reperfusion injury, Urine proteome, Animal model, LC–MS/MS

## Abstract

Mild hypothermia (MH) is an effective measure to alleviate cerebral ischemia–reperfusion (I/R) injury. However, the underlying biological mechanisms remain unclear. This study set out to investigate dynamic changes in urinary proteome due to MH in rats with cerebral I/R injury and explore the neuroprotective mechanisms of MH. A Pulsinelli’s four-vessel occlusion (4-VO) rat model was used to mimic global cerebral I/R injury. Liquid chromatography-tandem mass spectrometry (LC–MS/MS) was employed to profile the urinary proteome of rats with/without MH (32 °C) treatment after I/R injury. Representative differentially expressed proteins (DEPs) associated with MH were validated by western blotting in hippocampus. A total of 597 urinary proteins were identified, among which 119 demonstrated significant changes associated with MH. Gene Ontology (GO) annotation of the DEPs revealed that MH significantly enriched in endopeptidase activity, inflammatory response, aging, response to oxidative stress and reactive oxygen species, blood coagulation, and cell adhesion. Notably, changes in 12 DEPs were significantly reversed by MH treatment. Among them, 8 differential urinary proteins were previously reported to be closely associated with brain disease, including NP, FZD1, B2M, EPCR, ATRN, MB, CA1and VPS4A. Two representative proteins (FZD1, B2M) were further validated by western blotting in the hippocampus and the results were shown to be consistent with urinary proteomic analysis. Overall, this study strengthens the idea that urinary proteome can sensitively reflect pathophysiological changes in the brain, and appears to be the first study to explore the neuroprotective effects of MH by urinary proteomic analysis. FZD1 and B2M may be involved in the most fundamental molecular biological mechanisms of MH neuroprotection.

**Graphical Abstract:**

In a 4-VO rat model, 119 urinary proteins demonstrated significant changes associated with MH. MH is enriched in endopeptidase activity, inflammatory response, oxidative stress, etc, and significantly reversed changes in 12 DEPs. FZD1 and B2M are thought to be involved in the most fundamental molecular biological mechanisms of MH neuroprotection.
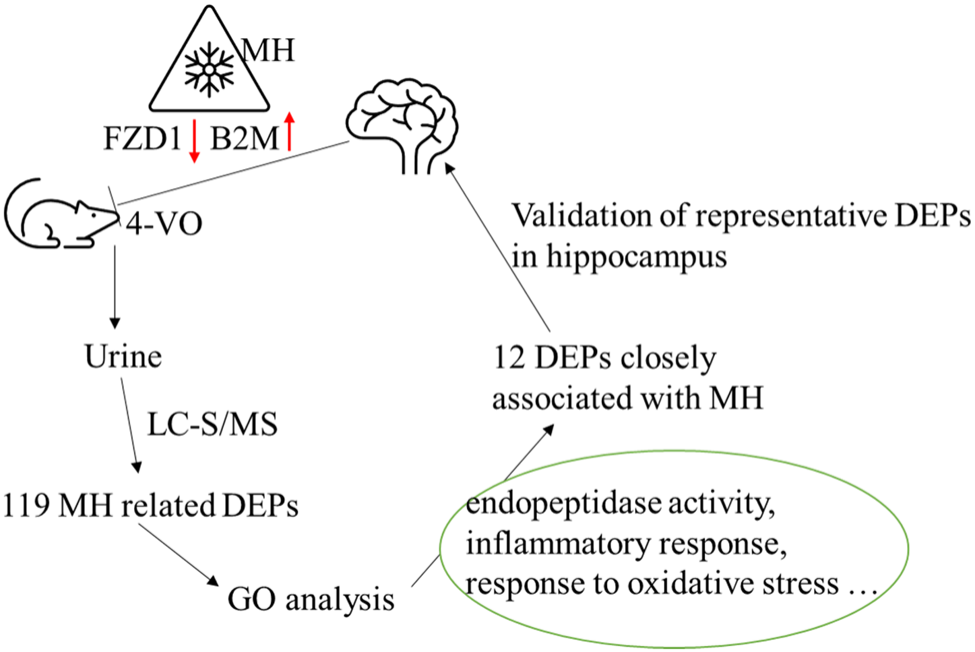

**Supplementary Information:**

The online version contains supplementary material available at 10.1007/s10571-024-01483-4.

## Introduction

Global cerebral ischemia–reperfusion (I/R) injury is the leading cause of death and poor quality of life, in severe hypotension caused by cardiac arrest and cardiopulmonary resuscitation (Aronowski et al. 1997; Yang and Betz 1994). Clinal studies have proved that mild hypothermia (MH) ameliorated cerebral I/R injury and improved neurological outcomes (Yenari and Han 2012). MH may alleviate I/R injury and improve the poor prognosis by affecting pathways of inflammation and oxidative stress, as well as metabolism and blood–brain barrier integrity (Zhao et al. 2018). It also influences angiogenesis, gliogenesis, and neurogenesis after cerebral I/R injury (Polderman 2009). However, the underlying mechanism of MH neuroprotection has not been clearly determined. An in-depth understanding of the molecular mechanisms of MH may be conducive to the exploration and development of new neuroprotective strategies.

Body fluids (blood, urine, cerebrospinal fluid, etc.) are important specimens for the study of neurological diseases biomarkers. However, the collection methods of blood and cerebrospinal fluid are invasively which limits the repeatability and reproducibility of detection. While urine could be collected non-invasively and continuously at large-scale, which is more easily accepted by patients. According to the largest human urine proteome database, over 30% of urinary proteins were enriched in brain tissue, ranking first among the 32 tissues and organs (Zhao et al. 2017). Hundreds of urinary proteins have been reported to be closely associated with a series of neurological diseases, such as, cerebrovascular disease, neurodegenerative disease and nerve tumors (An and Gao 2015). Our previous study revealed that urinary proteins could reflect the progression of cerebral I/R injury in rat model, when there were no clinical manifestations or histopathological cerebral damage (Sun et al. 2022). Overall, urine can sensitively reflect pathophysiological changes in the brain even at an early stage.

Mass spectrometry (MS)-based proteomics is a powerful tool that provides assessments with thousands of proteins investigated simultaneously. Recently, a couple of studies have deeply profiled the changes of brain tissue and plasma proteome after cerebral I/R injury (Datta et al. 2011; Kyng et al. 2018; Liu et al. 2020). Our previous study reported the neuroprotective effects of MH on the hippocampal proteome of experimental rat models of cerebral I/R injury (Wang et al. 2023). These findings provide an ample reserve of therapeutic targets and/or biomarker candidates for subsequent research. In the present study, we established a rat model of global cerebral I/R injury via Pulsinelli’s four-vessel occlusion (4-VO) method, and then label-free liquid chromatography-tandem mass spectrometry (LC–MS/MS) and bioinformatics analysis were used to determine the changes in urinary proteins associated with MH treatment. We aimed to elucidate the possible role of MH in the molecular pathways after cerebral I/R injury.

## Methods

### Animal Care and Experiments

Male SPF Wistar rats (8 weeks, 230 ± 20 g) purchased from Charles River Laboratories (Beijing, China) were used in this study. The animals were housed at constant room temperature on a light–dark cycle (12:12 h). All animals received humane care according to NIH guidelines. All experimental procedures and animal care were approved by the Animal Policy and Welfare Committee of Qingdao Municipal Hospital (2021Y39).

The rat model was established using Pulsinelli’s 4-VO method as previously described (Pulsinelli and Buchan 1988). Thirty rats were randomly divided into the following three groups: sham group, normothermia cerebral IR group, and MH cerebral IR group. The animals were anesthetized with 1% pentobarbital sodium (40 mg/kg). To establish the 4-VO rat model, the bilateral pterygoid foramens were surgically exposed, the bilateral vertebral arteries were electrocauterized, and the bilateral common carotid arteries were exposed. After 24 h, the common arteries were occluded with artery clamps (15 min). The rats lost the righting reflex within 30 s, and those unresponsive to light with dilated pupils were selected for the subsequent experiments. For rats in the sham group, the vessels were exposed without occlusion.

MH (32 ± 0.5 °C) was induced at the beginning of ischemia and maintained for 4 h. Core body temperature was monitored continuously using temporalis muscle and rectal temperature probes. An ice blanket was placed over the dorsum of prone rats until a body temperature of 32 ± 0.5 °C was achieved. The rats were then allowed to gradually rewarm back to their baseline temperature (37 ± 0.5 °C) during a 1-h period using heating lamps.

### Histological Analysis

The animals were anesthetized 24 h after the I/R injury and then they were perfused with 200 mL saline solution to flush the blood by left ventricular cannulation. Perfusion fixation was performed using 200 mL 4% paraformaldehyde (pH 7.2–7.4). After complete perfusion, the whole brains were harvested and fixed in fresh 4% paraformaldehyde at 4 °C. The fixed brain tissues were embedded in paraffin, and sectioned at 4 μm thickness. Sections were stained with hematoxylin and eosin (H&E) staining to reveal histopathological lesions.

### Urine Sample Preparation

Urine samples were collected for 4 h using metabolic cages. After collection, the urine were centrifuged at 12000×*g* for 30 min at 4 °C. After removing the pellets, four volumes of prechilled acetone were added to 1 mL urine and precipitated at 4 °C overnight. Lysis buffer (8 mol/L urea, 2 mol/L thiourea, 50 mmol/L Tris, and 25 mmol/L DTT) was used to redissolve the pellets. The protein concentration was measured by the Bradford protein assay. Urinary proteins were digested by 10-kDa filter aided trypsin (Sun et al. 2022). Briefly, 50 µg protein was reduced using dithiothreitol (DTT) (4.5 mM) for 1 h at 37 °C. It was then alkylated by mixing indoleacetic acid (10 mM) for 30 min at room temperature in the dark. The proteins were digested with trypsin (enzyme-to-protein ratio of 1:50) for 14 h at 37 °C. The peptide samples were subsequently lyophilized for LC–MS/MS analysis.

### LC–MS/MS Analysis

The peptide samples were analyzed using the Orbitrap Fusion Lumos Tribrid mass spectrometer coupled with EASY-nLC 1000 HPLC system (Thermo Scientific, Germany) as described previously (Qin et al. 2021; Sun et al. 2022). Briefly, the digested peptides were loaded onto a C18 trap column (3 µm, 75 µm × 2 cm, 100 A°) and then transferred to a C18 reversed-phase analytical column (2 µm, 50 µm × 250 mm, 100 A°). The elution acetonitrile gradient was maintained at 350 nL/min (5% to 30%) for 90 min. The calibration kit (iRT kit from Biognosys, Switzerland) reagent was spiked at a concentration of 1:20 v/v in all samples, in order to enable fully automated and sensitive signal processing. The same LC settings were used for DDA MS and DIA MS modes and the details of data-dependent acquisition (DDA) and data-independent acquisition (DIA) methods have been used as described previously (Sun et al. 2022). Briefly, the MS parameters were as follows: full scan from 350 to 1550 m/z at 120 000, cycle time of 3 s set to top speed mode, automatic gain control (AGC) of 1E6, and maximum injection time of 100 ms. MS2 scans were acquired with an isolation window of 2 Da at a resolution of 30 000 and 32% high-energy collision-induced dissociation (HCD). The AGC target was 5E5, and maximum injection time was 50 ms. (URL: https://www.iprox.cn/page/SSV024.html;url=1707898714294BYRU, passwords: 3aBn).

### Data Processing

The raw data files were processed using Proteome Discoverer (Thermo Scientific, San Jose, CA. version 2.1) and Spectronaut Pulsar as described previously (Sun et al. 2022). Following were the search parameters: SwissProt Rattus database (containing 8086 sequences), trypsin digestion, 10 ppm parent ion mass tolerance, 0.02 Da fragment ion mass tolerance, fixed modification carbamidomethylated cysteine (+ 58.00 Da), variable modifications of oxidized methionine (+ 15.995 Da), and deamidated glutamine asparagine (+ 0.984 Da). The other parameters were set as default. After normalization, peptide intensity was calculated by summing the peak areas of their respective fragment ions for MS2. A false discovery rate (FDR) was set to 0.01 at protein level. The DEPs were selected using one-way ANOVA. Significance was set at 1.5-fold change and *p* < 0.05.

### Bioinformatics Analysis

The Database for Annotation, Visualization, and Integrated Discovery (DAVID) 6.8 (https://david.ncifcrf.gov/) was used to analyze the functional annotation of the differential proteins (The Gene Ontology Resource: 20 years and still GOing strong 2019) (Ashburner et al. 2000). In this study, significant GO enrichment was defined as *p* < 0.05. Protein–protein interaction (PPI) networks were constructed using the STRING database (http://www.string-db.org), which is a database of known and predicted protein interactions, including direct (physical) and indirect (functional) associations.

### Western Blot Analysis

Hippocampus proteins (30 μg) were separated by SDS-PAGE and transferred to polyvinylidene difluoride membranes. After blocking in TBST buffer (1X TBS containing 0.1% Tween 20) with 5% (w/v) skimmed milk for one hour at room temperature, the membranes were incubated with diluted primary antibodies (Anti-FZD1 antibody, 1:1000, Santa Cruz, INC, US, catalog #: sc-398082, PMID:8626800; Anti-B2M antibody, 1:4000, Abcam, Cambridge, UK, catalog #: Ab75853, PMID: 34667030; Anti-β-actin antibody, Abcam, Cambridge, UK, catalog #: ab179467, PMID:34533236) with gentle shaking overnight at 4 °C. After washing three times with TBST buffer, the membrane was probed with HRP-conjugated secondary antibodies (1: 10000, Biosharp, catalog #: BL003A, PMID: 32117963; 1: 10000, Affinity, catalog #: S0002, PMID:31962167) coupled to horseradish peroxidase at room temperature for one hour. Densitometry analysis was performed using ImageJ software, *p*-values were calculated using one-way ANOVA.

Each experiment and the statistical calculations described below were carried out in a randomized order by the experimenter blinded to the group.

## Results and Discussion

### Attenuation of Neurological Damage by MH After Cerebral I/R Injury

**Fig. 1:**
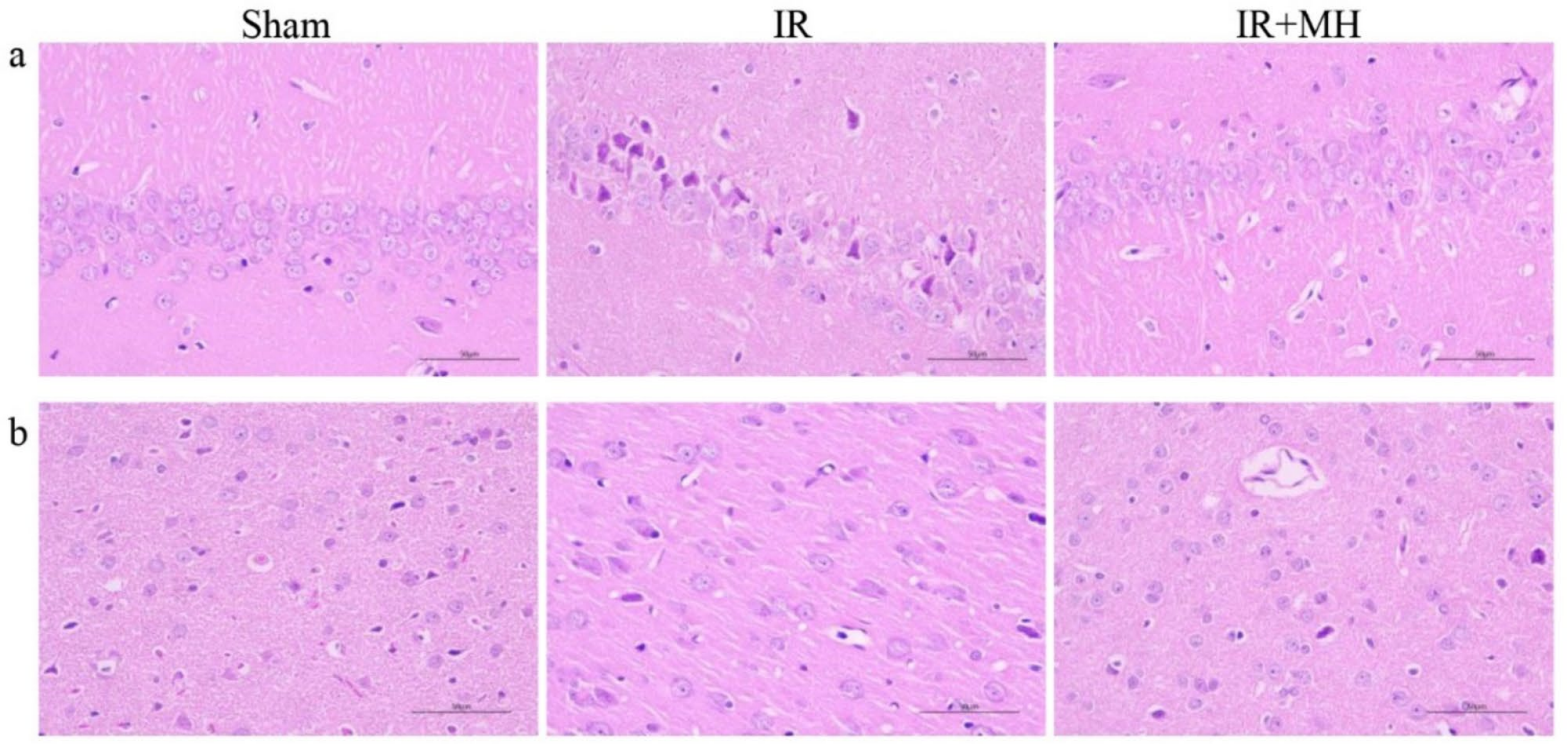
Protective effects of mild hypothermia on neurological damage after cerebral ischemia–reperfusion (IR) injury. **a** Hippocampal CA1 region, **b** Cerebral cortex. *Sham* Sham-operation group, *IR* cerebral I/R injury followed by normothermia (37 °C) group, *IR + MH* cerebral I/R injury followed by 4 h of MH (32 °C) group. Scale bars = 50 µm

To examine the histological damage due to ischemia and the neuroprotective effect of MH, HE staining of the cerebral cortex and hippocampus was performed. It showed that the structure of the hippocampal CA1 region neurons in the sham group was complete, and the cell arrangement and morphology were normal. The number of neurons was reduced, the gaps were widened, and typical apoptotic cells (shrunken cell bodies and nuclear pyknosis) were observed in the IR group. The morphology of nerve cells demonstrated improvement with MH treatment (Fig. 1a). HE staining of the cerebral cortex revealed no neuron morphology abnormalities in the sham group. The IR group showed edema of the neurons and some cellular structures were incomplete. MH was markedly found to ameliorate pathological changes due to IR (Fig. 1b).

### Profiling of Urinary Proteome After Cerebral I/R Injury With/Without MH

MH is an effective therapeutic strategy to reduce neuronal injury after cerebral I/R. However, the specific mechanism by which it mediates neuroprotective signaling pathways is still unclear. Using a quantitative proteomics protocol, we performed global profiling of the urinary proteins of rats, which were subjected to sham or 4-VO operation, followed by 4 h of MH or normothermia (*n* = 7 per group).

**Fig. 2:**
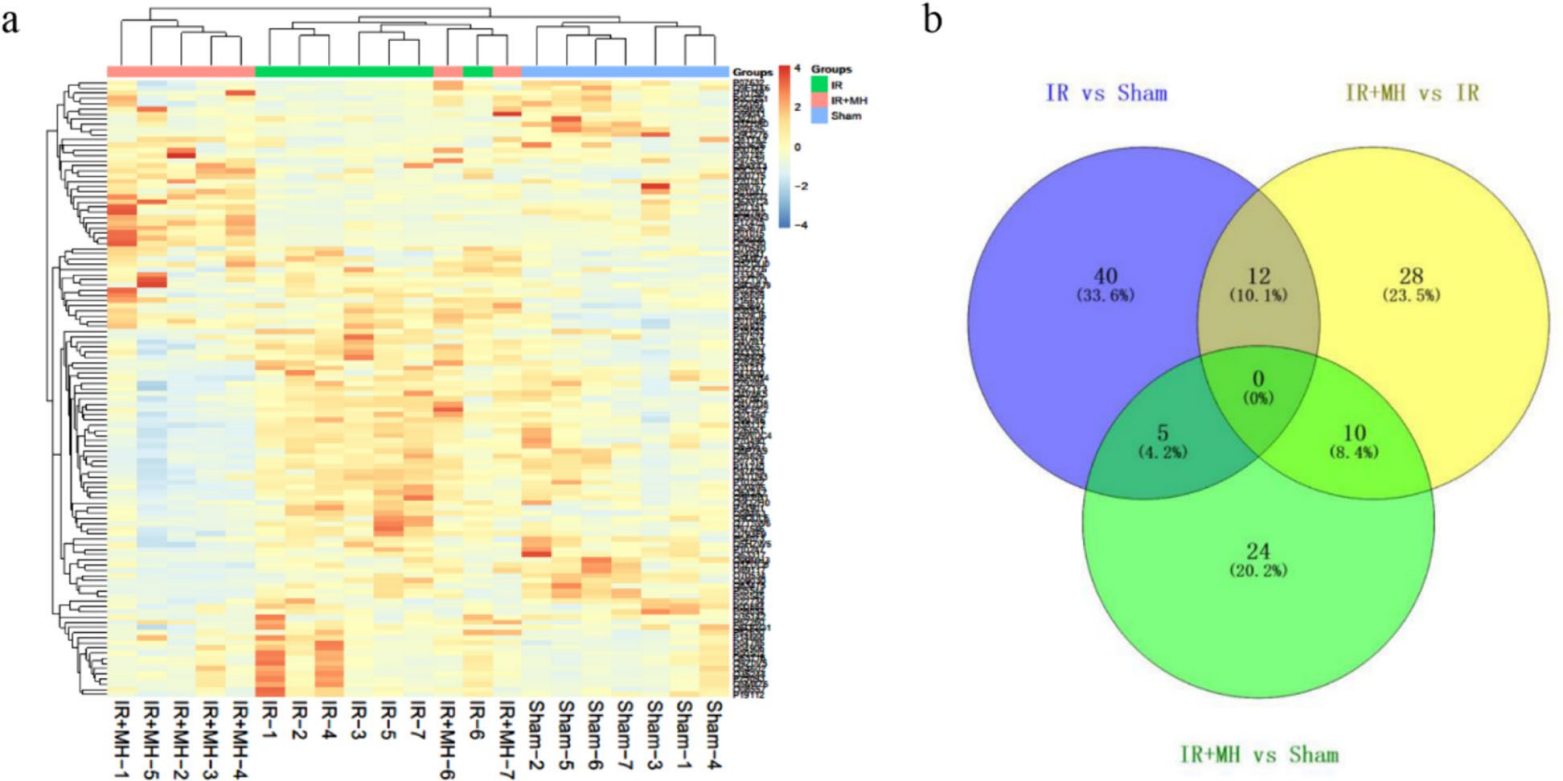
Differentially expressed urinary proteins after cerebral ischemia–reperfusion (IR) injury followed by normothermia or MH. **a** Hierarchical cluster analysis, **b** Venn diagram. *Sham* Sham-operation group, *IR* cerebral I/R injury followed by normothermia (37 °C) group, *IR + MH* cerebral I/R injury followed by 4 h of MH (32 °C) group

After LC–MS/MS analysis, 597 urinary proteins were identified with at least one unique peptide (FDR < 1%). Among these, 119 proteins were significantly changed (fold change-1.5, *p* < 0.05). The quantification of DEPs is shown in Table S1. The hierarchical cluster analysis of DEPs showed that MH could significantly reverse expression trends of proteins due to IR (Fig. 2a). After cerebral I/R injury, 57 urinary proteins were changed when compared with the sham controls, with 41 proteins being upregulated and 16 proteins, downregulated. With MH treatment, 39 urinary proteins were changed when compared with the sham controls, with 13 proteins being upregulated and 26 proteins, downregulated. MH changed 50 urinary proteins after cerebral I/R injury, with 15 proteins being upregulated and 35, downregulated. The overlap of the DEPs identified between different groups is shown as a Venn diagram (Fig. 2b and Table S1).

### Function Annotation Analysis of DEPs Associated with MH

**Fig. 3:**
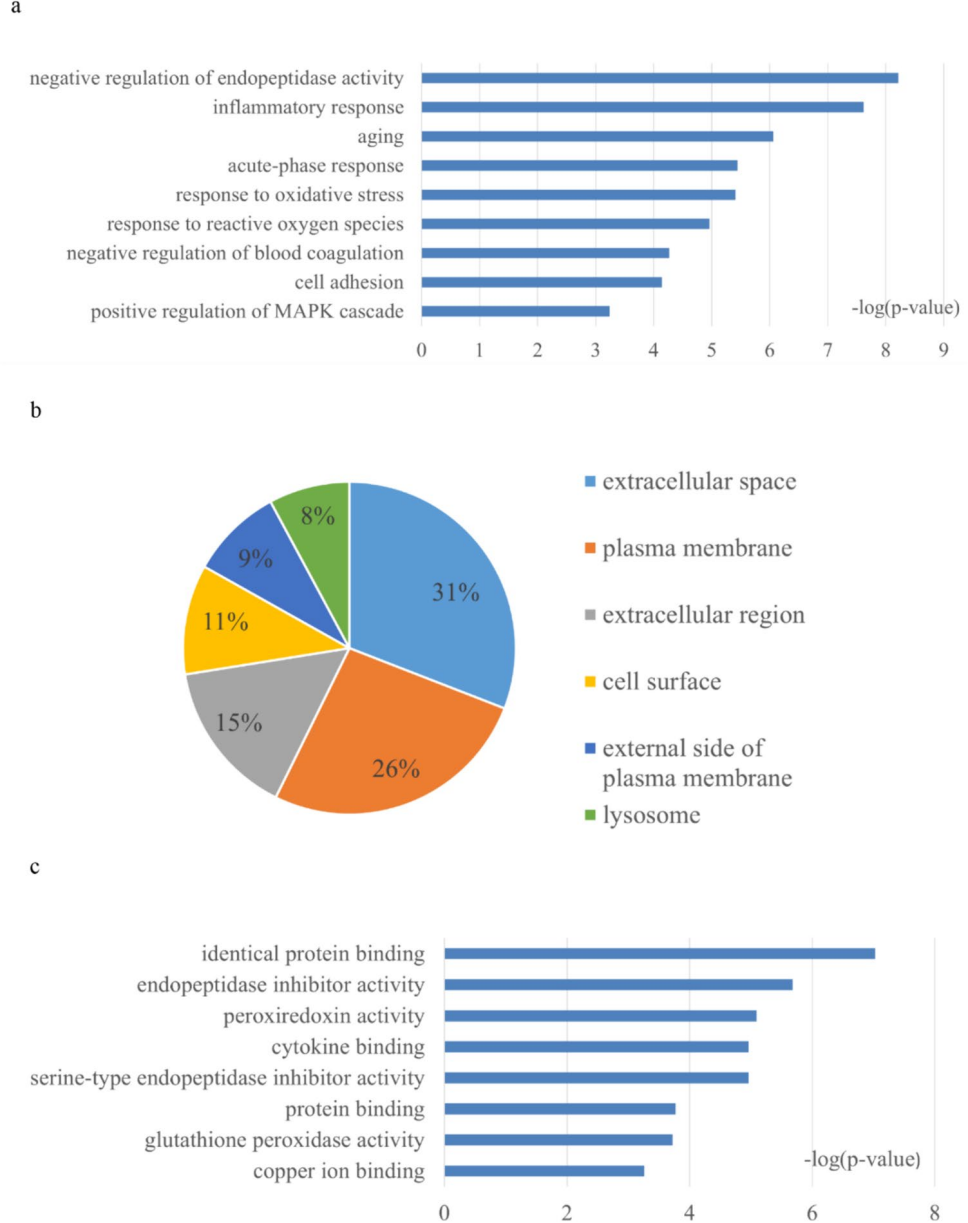
GO analysis of the DEPs associated with mild hypothermia. **a** Biological process, **b** Cellular component, **c** Molecular function; (*p* value < 0.05)

The functional annotation of the 119 DEPs consisted of sorting them into the following categories using DAVID: biological process, cellular component, and molecular function. In the biological process category, negative regulation of endopeptidase activity, inflammatory response, aging, acute-phase response, response to oxidative stress, response to reactive oxygen species, negative regulation of blood coagulation, cell adhesion, and positive regulation of mitogen-activated protein kinase (MAPK) cascade were overrepresented with MH treatment (Fig. 3a). In the cellular component category, most of these DEPs were from the extracellular space, extracellular region, lysosome, cell surface, and external side of the plasma membrane (Fig. 3b). In the molecular function category, identical protein binding, endopeptidase inhibitor activity, peroxiredoxin activity, serine-type endopeptidase inhibitor activity, cytokine binding, protein binding, glutathione peroxidase activity, and copper ion binding were overrepresented with MH treatment (Fig. 3c).

### Protein–Protein Interactions of DEPs Associated with MH

**Fig. 4:**
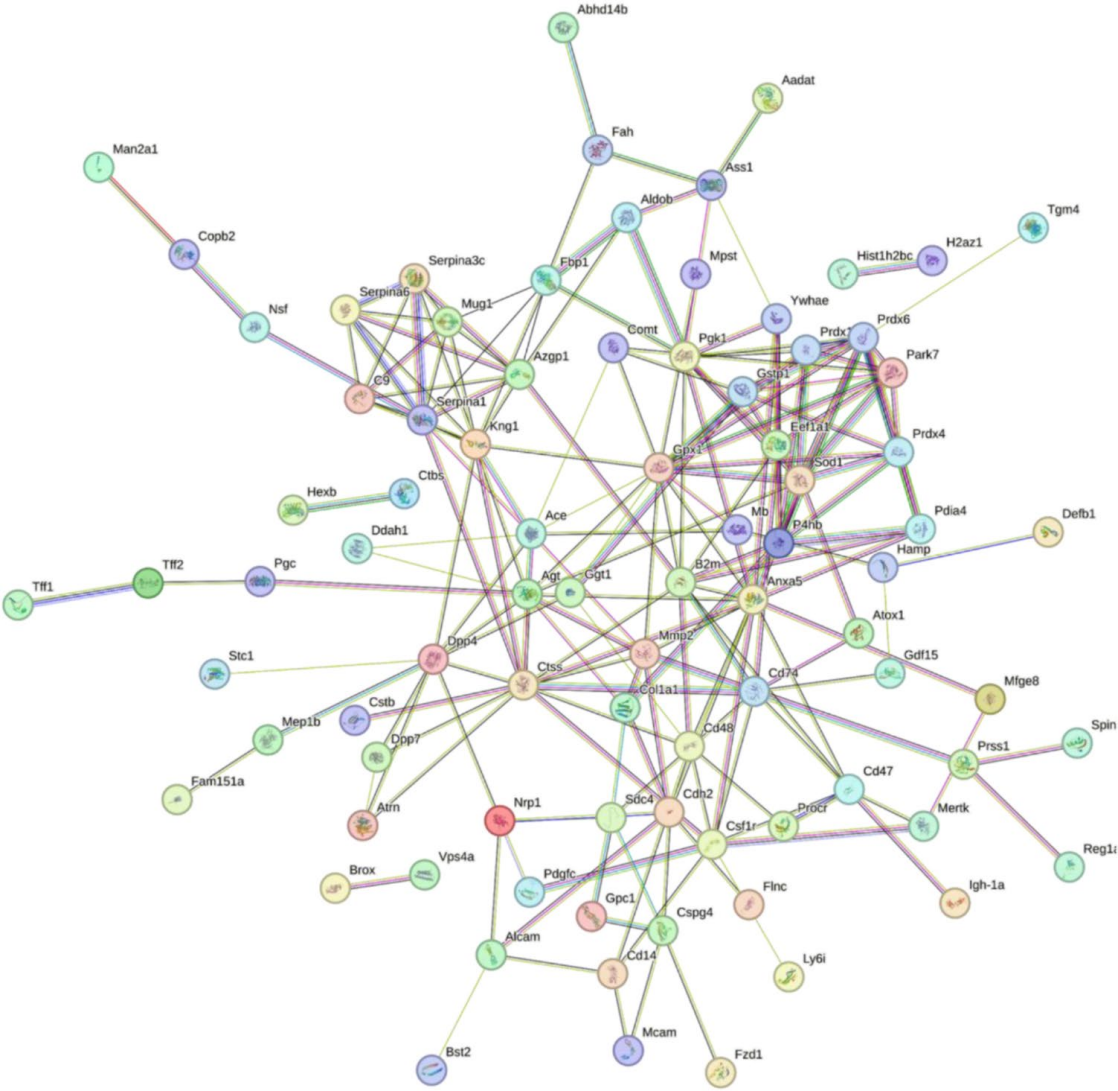
STRING protein–protein interaction network analysis of differentially expressed proteins associated with mild hypothermia. The number of nodes is 112, the average node degree is 3.54, and the average local clustering coefficient is 0.394 (*p*-value < 1.0e−16)

To better understand the neuroprotective mechanisms of MH, a PPI network for 119 DEPs was constructed using STRING (Fig. 4). The STRING PPI network analysis showed that the average node degree was 3.54, the average local clustering coefficient was 0.394, and the PPI enrichment *p* value < 1.0e−16. The above results show that these DEPs had more interactions among themselves than what is expected for a random set of proteins of similar size.

### Urinary DEPs Closely Associated with MH Neuroprotection

The expression trends of the 12 DEPs were reversed by MH (Table 1). Nine urinary DEPs were highly expressed in rats with cerebral I/R injury and significantly reversed by MH treatment, while the other three DEPs displayed low expression in rats with I/R injury and then high expression with MH treatment. Among them, eight urinary proteins, including neuroplastin (NP), frizzled-1 (FZD1), endothelial protein C receptor (EPCR), vacuolar protein sorting-associated protein 4A (VPS4A), attractin (ATRN), myoglobin (MB), carbonic anhydrase 1 (CA1), and beta-2-microglobulin (B2M), have been shown to be involved in brain disease, such as hypoxic-ischemic encephalopathy, Alzheimer’s disease, and cerebral tumors (Table 1). Additionally, two proteins (FZD1, B2M) are known to be related to neuroinflammation and cerebral ischemia. The remaining four proteins, including syndecan-4 (SDC4), C-type mannose receptor 2 (MRC2), protein FAM151A, store-operated calcium entry regulator STIMATE, were first reported to be associated with cerebral I/R injury and MH neuroprotection.

**Table 1:** The DEPs in urine closely associated with mild hypothermia

Uniprot ID	Protein name	Human ortholog	IR vs Sham		IR + MH vs IR		Diseases’ biomarkers
			FC	*p* value	FC	*p* value	
P34901	Syndecan-4	P31431	2.50	0.008	2.86	0.010	–
P97546	Neuroplastin	Q9Y639	1.80	0.034	2.55	0.042	Alzheimer's disease (Ilic et al. (2018)
Q08463	Frizzled 1	Q9UP38	2.35	0.010	2.13	0.044	Cerebral ischemia, apoptosis Mardones et al. (2016), Matei et al. (2018)
Q4TU93	C-type mannose receptor 2	Q9UBG0	1.82	0.007	1.95	0.028	–
Q4V8I1	Endothelial protein C receptor	Q9UNN8	1.83	0.018	2.08	0.018	Gliomas Berindan-Neagoe et al. (2012)
Q642A7	Protein FAM151A	Q8WW52	1.56	0.050	2.78	0.027	–
Q793F9	Vacuolar protein sorting-associated protein 4A	Q9UN37	1.73	0.015	2.18	0.049	Structural brain abnormalities, neurodevelopmental delay Rodger et al. (2020)
Q7TSW6	Store-operated calcium entry regulator STIMATE	Q86TL2	2.84	0.043	6.58	0.009	–
Q99J86	Attractin	O75882	1.78	0.001	1.81	0.043	Inflammation Li et al. (2021),learning and memory Liu et al. (2023)
Q9QZ76	Myoglobin	P02144	− 9.51	0.047	3.88	0.014	brain cancers, hypoxia Elsherbiny et al. (2021; El‑Tohamy et al. 2020)
B0BNN3	Carbonic anhydrase 1	P00915	− 9.46	0.030	8.44	0.004	neuropsychiatric disorders Cheng et al. (2023)
P07151	Beta-2-microglobulin	P61769	− 2.70	0.028	4.94	0.035	hypoxic-ischemic encephalopathy Carreras et al. (2022), Alzheimer’s disease Zhao et al. (2023), cerebral tumor(Li et al. 2022)

“–” means down-regula

### Validation of DEPs Associated with MH by Western Blotting in *Hippocampus*

**Fig. 5:**
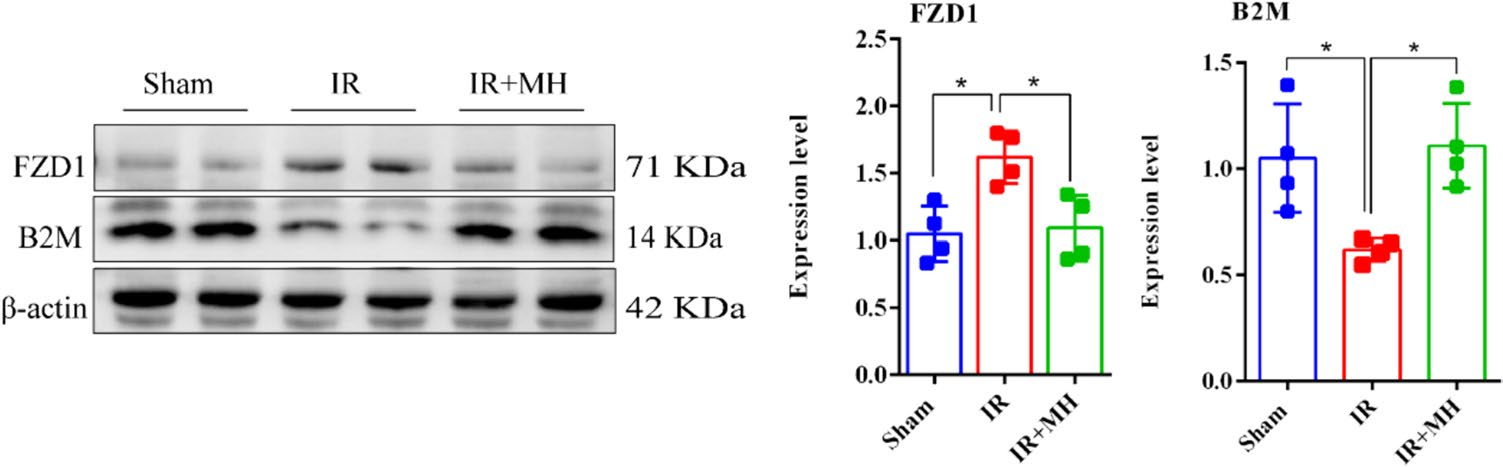
Validation of two DEPs associated with MH by western blotting. **a** Representative blots visualizing the levels of FZD1 and B2M in the three groups. β-actin was used as the loading control. *n* = 4. **b** Quantitative analysis of the expression level of FZD1 and B2M in the hippocampus. The data passed normality test. FZD1, One way ANOVA, F (2, 9) = 8.683, *p* = 0.0079; Brown–Forsythe test, F (DFn, DFd) = 0.6464 (2,9), *p* = 0.5466; Tukey’s multiple comparisons test, Sham vs. IR: *p* = 0.0116, IR vs. IR + MH: *p* = 0.0177. B2M, One way ANOVA, F (2,9) = 7.884, *p* = 0.0105; Brown–Forsythe test, F (DFn, DFd) = 1.537 (2,9), *p* = 0.2666; Tukey’s multiple comparisons test, Sham vs. IR: *p* = 0.0264, IR vs. IR + MH: *p* = 0.0136. (* p < 0.05)

According to the bioinformatic analyses of the DEPs, consulting relevant publications and commercially available reagents, two representative DEPs FZD1 and B2M which were closely associated with cerebral I/R injury were selected for further validation by western blotting in extended samples. The two proteins are included in protein–protein interaction network nodes (Fig. 4). The contents of the selected DEPs in the ipsilateral hippocampus samples were calculated. As expected, the western blot results of the two DEPs were consistent with the results of the proteomic analysis. Significantly different amounts of FZD1 (*p* < 0.05) and B2M (*p* < 0.05) were observed after cerebral I/R injury with and without MH treatment (Fig. 5). FZD1 was highly expressed in the cerebral I/R injury rats and significantly lowered by MH treatment. The expression of B2M displayed lower expression in the I/R injury rats and then elevated to basal levels with MH treatment.

## Discussion

In this study, we aimed to explore the effect of MH on urinary protein expression and the molecular mechanism of hypothermic neuroprotection after cerebral I/R injury. To the best of our knowledge, this is the first time, a systematic investigation of dynamic changes in urinary proteome was performed using a 4-VO rat model with/without MH based on proteomics analysis. A total of 597 proteins were identified (FDR < 1%). Among them, 119 were DEPs associated with MH treatment. Many of these DEPs were involved in endopeptidase activity, acute-phase inflammatory response, oxidative stress and response to reactive oxygen species, cell adhesion and blood coagulation, and MAPK cascade. Changes in 12 DEPs were reversed by MH treatment in the cerebral I/R injury group. Additionally, all these DEPs were found to have counterparts in humans (Table 1). These results suggest that human urine proteome may change to some extent owing to MH treatment. They also provide insight into the potential mechanisms of the neuroprotective effects of MH.

FZD1, the initial member of the frizzled gene family, encodes seven transmembrane proteins that function as receptors for the Wnt signaling pathway (Jia et al. 2023). In the context of hippocampal development, FZD1 exhibits varying levels of expression (Mardones et al. 2016). The wnt3a, a potential neuroprotective agent, can reduce infarction and enhance behavioral outcomes in rats with middle cerebral artery occlusion (MCAO) by mitigating neuronal apoptosis and promoting cell survival through the FZD1/PIWI1a/FOXM1 pathway (Matei et al. 2018). Moreover, Wu et al. conducted a study using a rat brain injury post subarachnoid hemorrhage model to demonstrate the antiapoptotic effects of intranasal wnt3a through the FZD/aldolase C/PPAN pathway (Ruan et al. 2020). Additionally, FZD1 has also been identified as a potential therapeutic target in traumatic spinal cord injuries (González et al. 2020). In our current investigation, we observed a significant upregulation of FZD1 in the urine of rats after cerebral I/R injury, which was subsequently reversed by MH treatment. These findings suggest that MH may exert a neuroprotective effect by reducing the expression of FZD1.

B2M, a prominent constituent of the major histocompatibility complex class I in plasma, assumes a critical function in the regulation of inflammatory responses. Within the central nervous system, B2M is involved in the pathogenesis of diverse cerebral disorders, such as encephalopathy (Carreras et al. 2022), Alzheimer’s disease (Zhao et al. 2023), and cerebral tumors (Li et al. 2022). The question of whether B2M exerts a beneficial or detrimental impact in the context of brain diseases remains a subject of controversy. While an elevation of B2M in infants with hypoxic ischemic encephalopathy has been demonstrated in one study (Carreras et al. 2022), another provided evidence that B2M induces M2-like macrophage polarization and alters the tumor immune microenvironment toward an anti-inflammatory state (Li et al. 2022). Furthermore, the implication of B2M in various other diseases has been suggested, as evidenced by the induction of synaptic and memory defects in mice with Down syndrome after the systemic administration of B2M. The genetic elimination of B2M or the systemic application of an anti-B2M antibody effectively mitigates synaptic impairments in mice with Down syndrome (Gao et al. 2023). The current study showed that the urinary concentration of B2M decreased by approximately three times after an injury induced by IR. However, upon treatment with MH, the B2M concentration surpassed the basal levels, suggesting the potential significance of B2M in the neuroprotective effects associated with MH. Consequently, additional research is required to comprehensively elucidate the mechanisms by which B2M supports neural functions in the context of MH.

The proteomic assessments conducted in our study revealed elevated expression levels of NP, ATRN, and VPS4A in rats with cerebral I/R injury. MH treatment effectively reversed these observed changes. Findings obtained from animal models provide evidence supporting the involvement of NP in pathways implicated in neuropsychiatric and neurodegenerative disorders. The loss or disruption of NP and its associated molecular pathways pertaining to neuronal processes is known to be associated with various neurological conditions, such as dementia, schizophrenia, and Alzheimer's disease (Lin et al. 2021). ATRN plays a crucial role in the initial aggregation of immune cells during inflammatory responses and potentially modulates the chemotaxis activity of chemokines. It is a glycoprotein with widespread expression that maintains energy homeostasis, facilitates neurodevelopment, and orchestrates immune responses (Li et al. 2021). The current findings show that ATRN mutations hinder myelination and adversely affect learning and memory in rats by inhibiting the BDNF/TrkB and Nrg‐1/ErbB4 signaling pathways (Liu et al. 2023). The proper functioning of VPS4A is essential for various human developmental and cellular processes. The occurrence of de novo VPS4A mutations results in a multi-system disorder characterized by abnormal neurodevelopment (Rodger et al. 2020). In a separate investigation, VPS4A knockout mice were employed to anticipate the effects of VPS4A deletion, which demonstrated a hindrance in autophagic flux, leading to the accumulation of degradation substances and compromised cardiac function (Huang et al. 2023). Despite extensive research regarding autophagy in cerebral I/R injury, studies pertaining to the involvement of VPS4A in regulating autophagic flux for this specific injury have not been conducted to date. The findings of this study indicate that NP, ATRN, and VPS4A could potentially serve as crucial endogenous protective proteins in the context of neuroprotection associated with MH. Nevertheless, further investigation is necessary to elucidate the precise mechanisms underlying the observed associations of these significantly expressed proteins due to MH induction in rats with cerebral I/R injury.

## Conclusion

We investigated the neuroprotective effects of MH on the urine proteome of experimental models of brain I/R injury. The general overview of protein regulation presented in our study provides insights into the potential neuroprotective mechanisms of MH. Additionally, we revealed the key DEPs, namely FZD-1, B2M, NP, ATRN, VPS4A, and verified two of them (FZD-1, B2M) in the hippocampus. These findings may provide a basis for the subsequent study of the mechanism of MH in neuroprotection and leading to new drug targets for cerebral I/R therapies.

## Supplementary Information

Below is the link to the electronic supplementary material.

Supplementary file1 (TIF 227 kb)

Supplementary file2 (TIF 227 kb)

Supplementary file3 (TIF 2977 kb)

Supplementary file4 (TIF 2977 kb)

Supplementary file5 (TIF 2977 kb)

Supplementary file6 (TIF 2977 kb)

Supplementary file7 (TIF 227 kb)

Supplementary file8 (TIF 534 kb)

Supplementary file9 (TIF 639 kb)

Supplementary file10 (XLSX 35 kb)

Supplementary file11 (XLSX 10 kb)

## Data Availability

The datasets used and/or analyzed during the current study are available from the corresponding author on reasonable request.

## Declarations

### Statement on ARRIVE Guidelines

We declared that this study was carried out in compliance with the ARRIVE guidelines.
