# Supplementary figures and images for: Urine Proteomic Signatures of Mild Hypothermia Treatment in Cerebral Ischemia–Reperfusion Injury in Rats
Source: Cell Mol Neurobiol. 2024 Jun 5;44:49. doi: 10.1007/s10571-024-01483-4 (PMC11153299; doi:10.1007/s10571-024-01483-4)

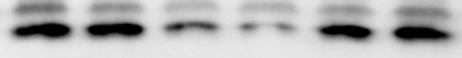

Supplement: Supplementary file 1 — Supplementary file1 (TIF 227 kb) [file 10571_2024_1483_MOESM1_ESM.tif]

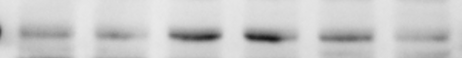

Supplement: Supplementary file 2 — Supplementary file2 (TIF 227 kb) [file 10571_2024_1483_MOESM2_ESM.tif]

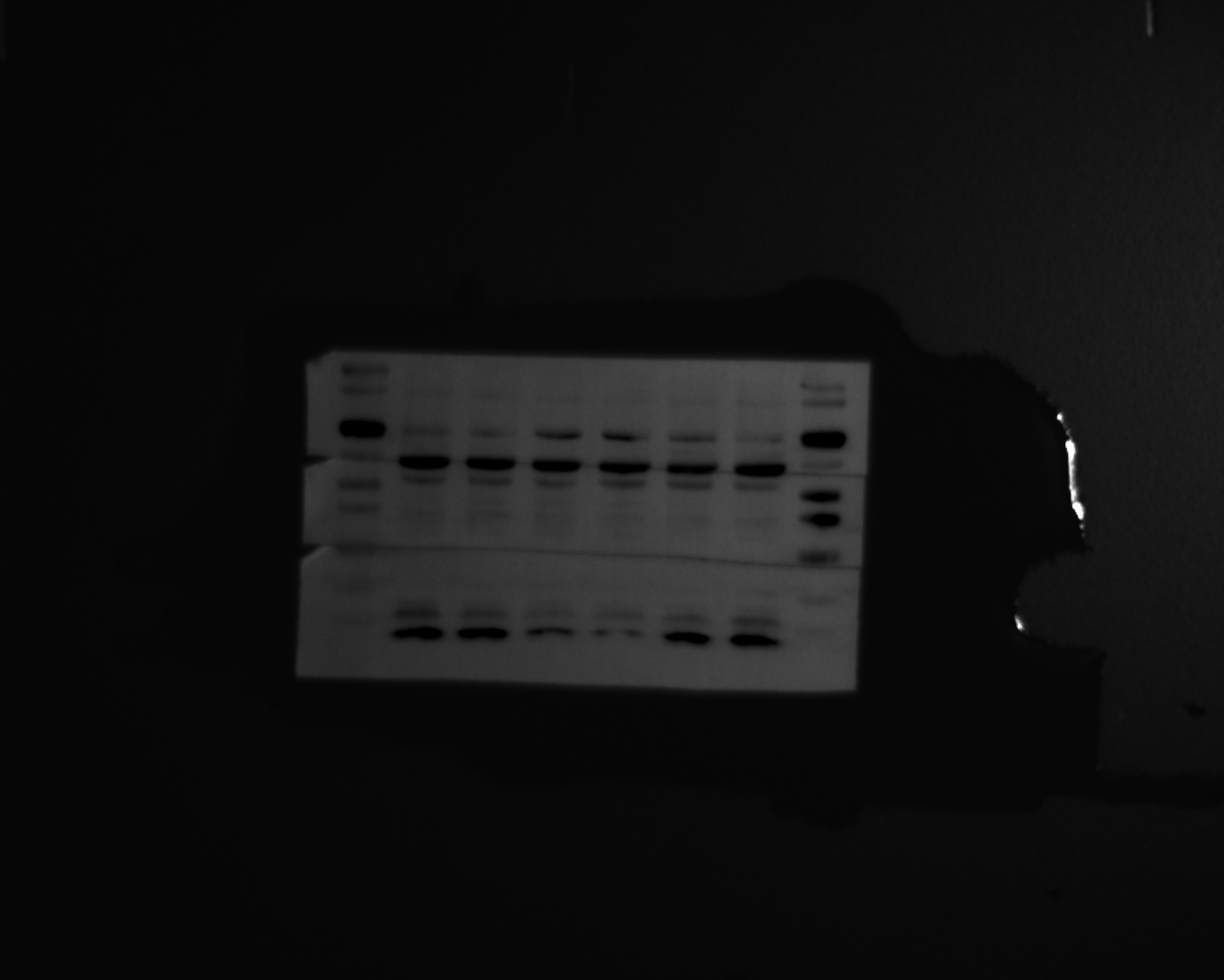

Supplement: Supplementary file 3 — Supplementary file3 (TIF 2977 kb) [file 10571_2024_1483_MOESM3_ESM.tif]

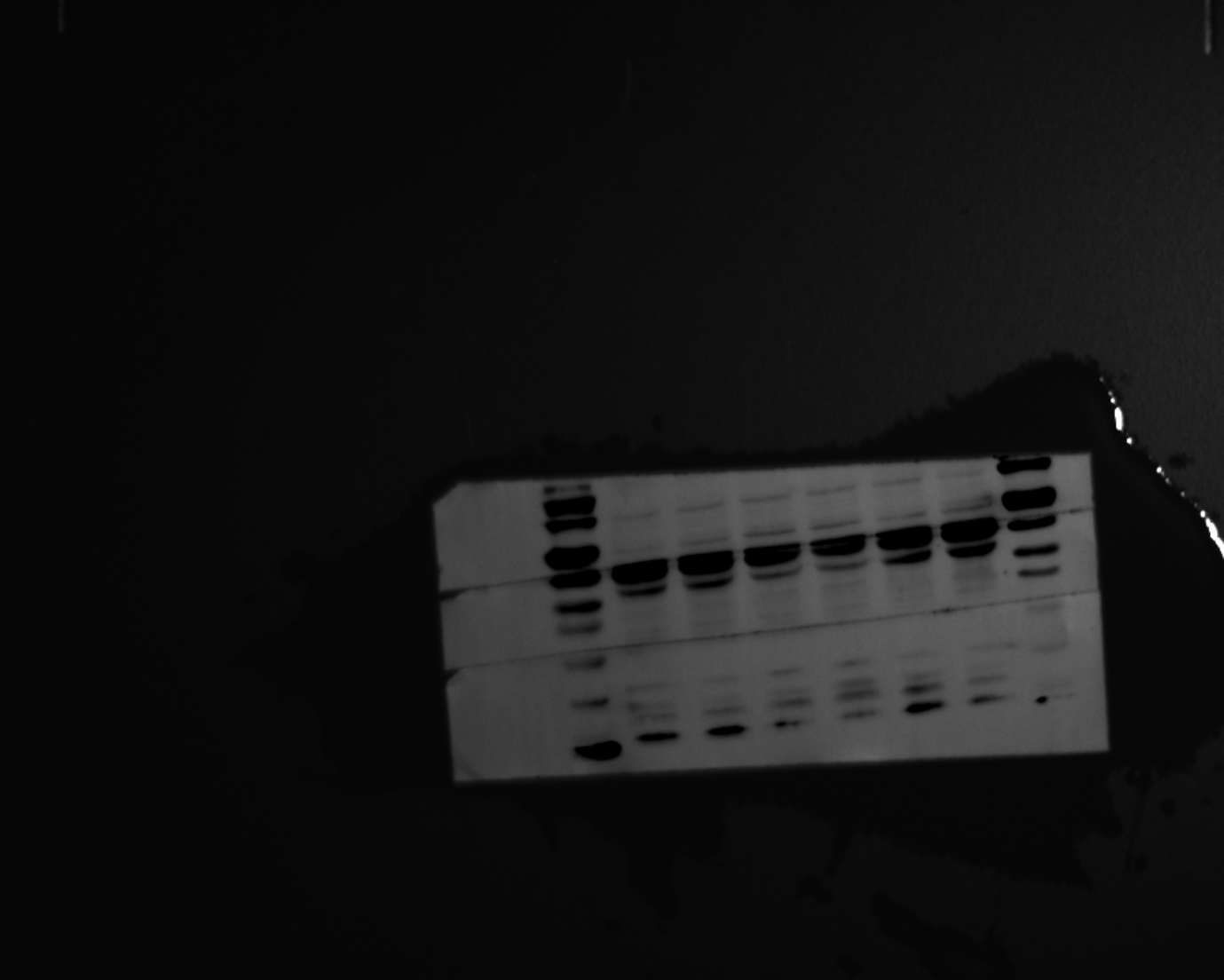

Supplement: Supplementary file 4 — Supplementary file4 (TIF 2977 kb) [file 10571_2024_1483_MOESM4_ESM.tif]

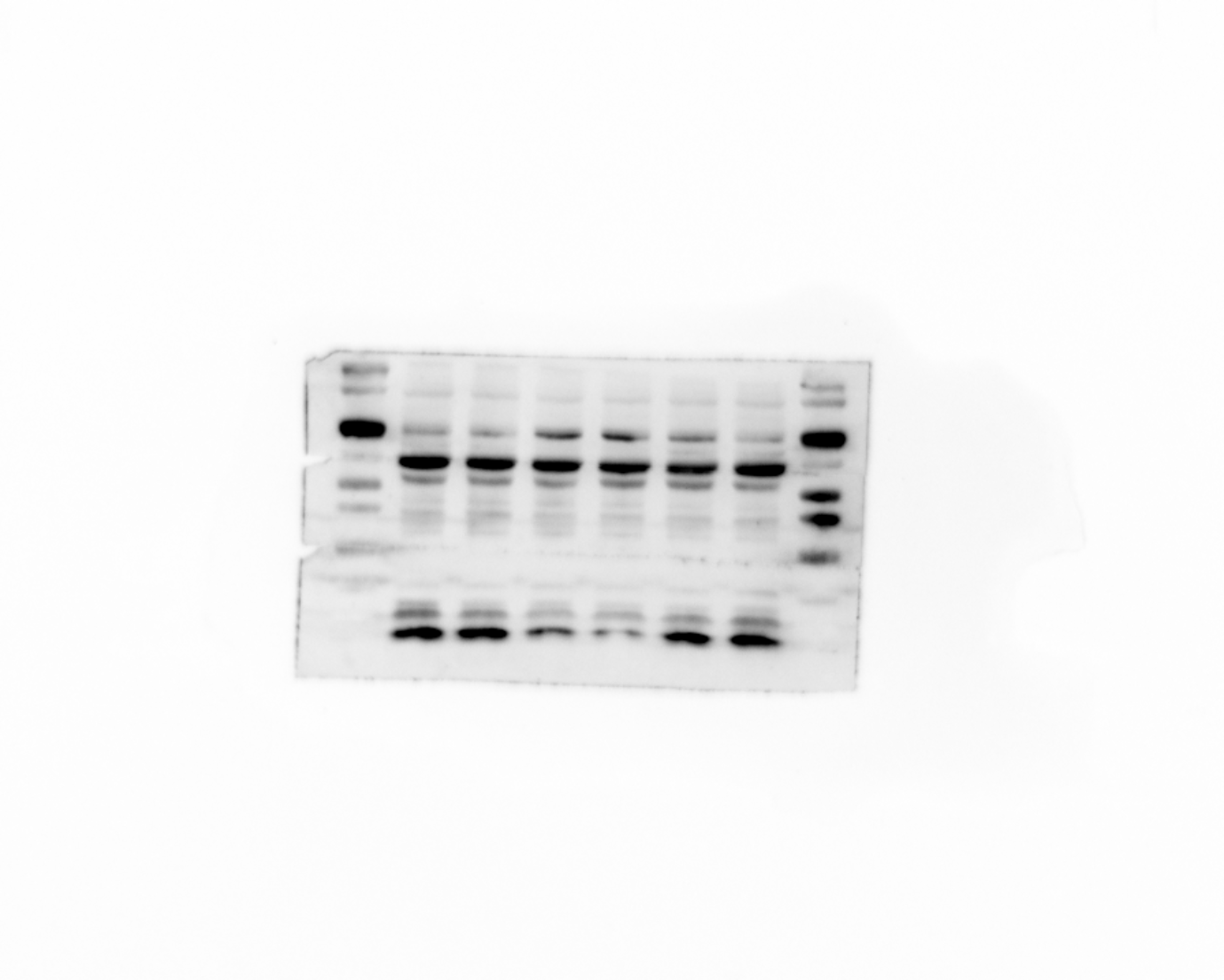

Supplement: Supplementary file 5 — Supplementary file5 (TIF 2977 kb) [file 10571_2024_1483_MOESM5_ESM.tif]

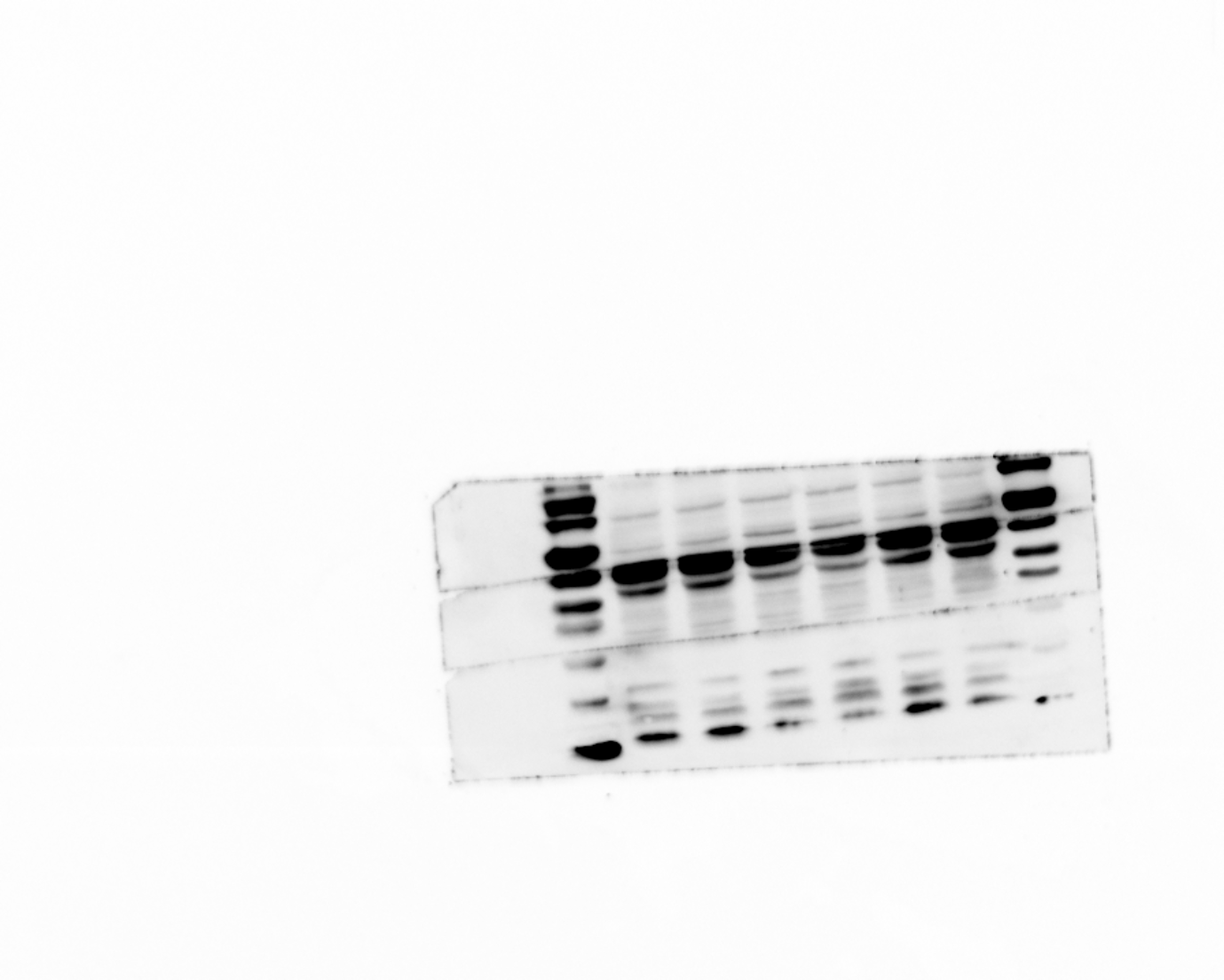

Supplement: Supplementary file 6 — Supplementary file6 (TIF 2977 kb) [file 10571_2024_1483_MOESM6_ESM.tif]

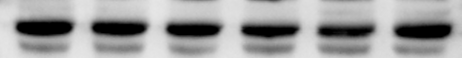

Supplement: Supplementary file 7 — Supplementary file7 (TIF 227 kb) [file 10571_2024_1483_MOESM7_ESM.tif]

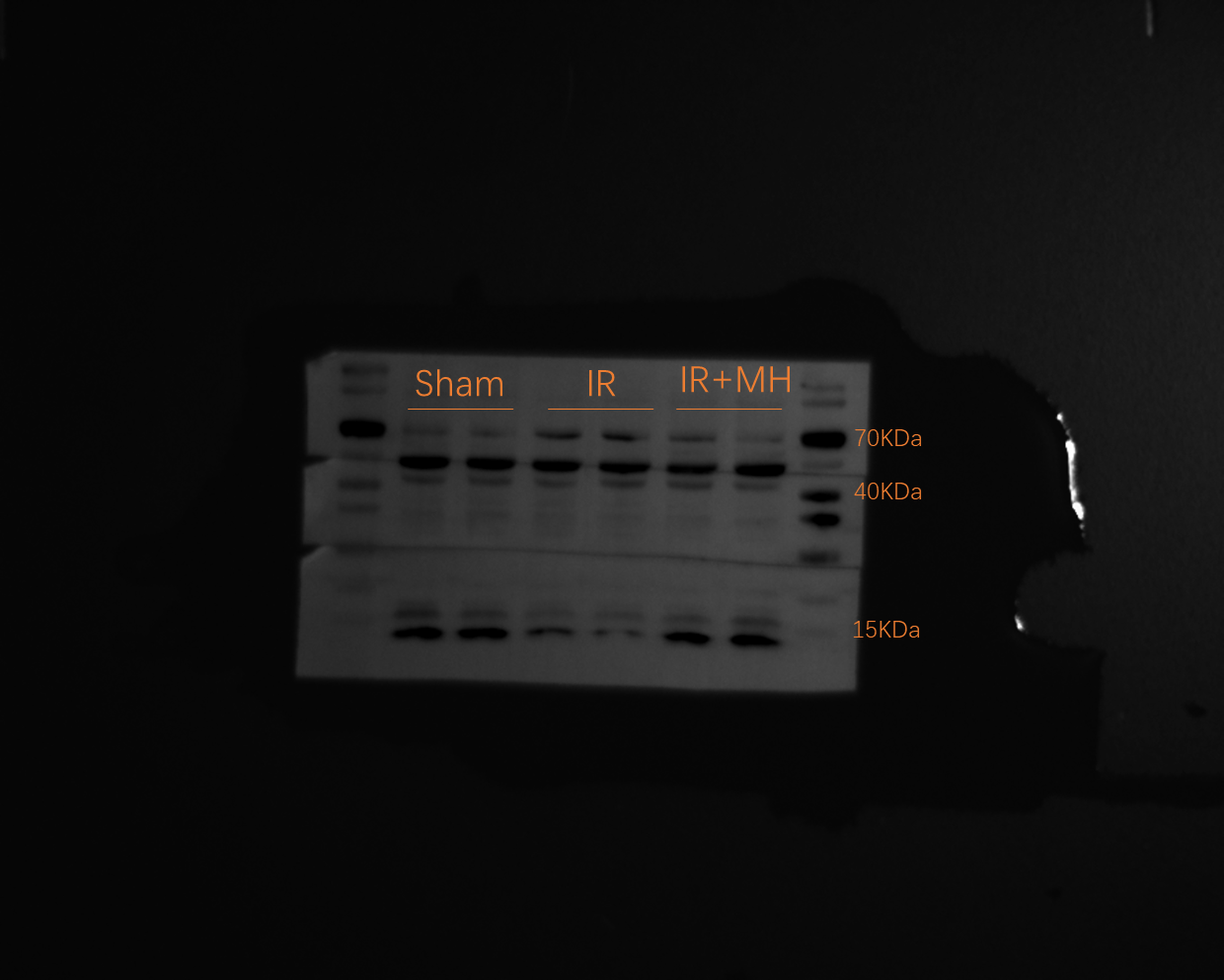

Supplement: Supplementary file 8 — Supplementary file8 (TIF 534 kb) [file 10571_2024_1483_MOESM8_ESM.tif]

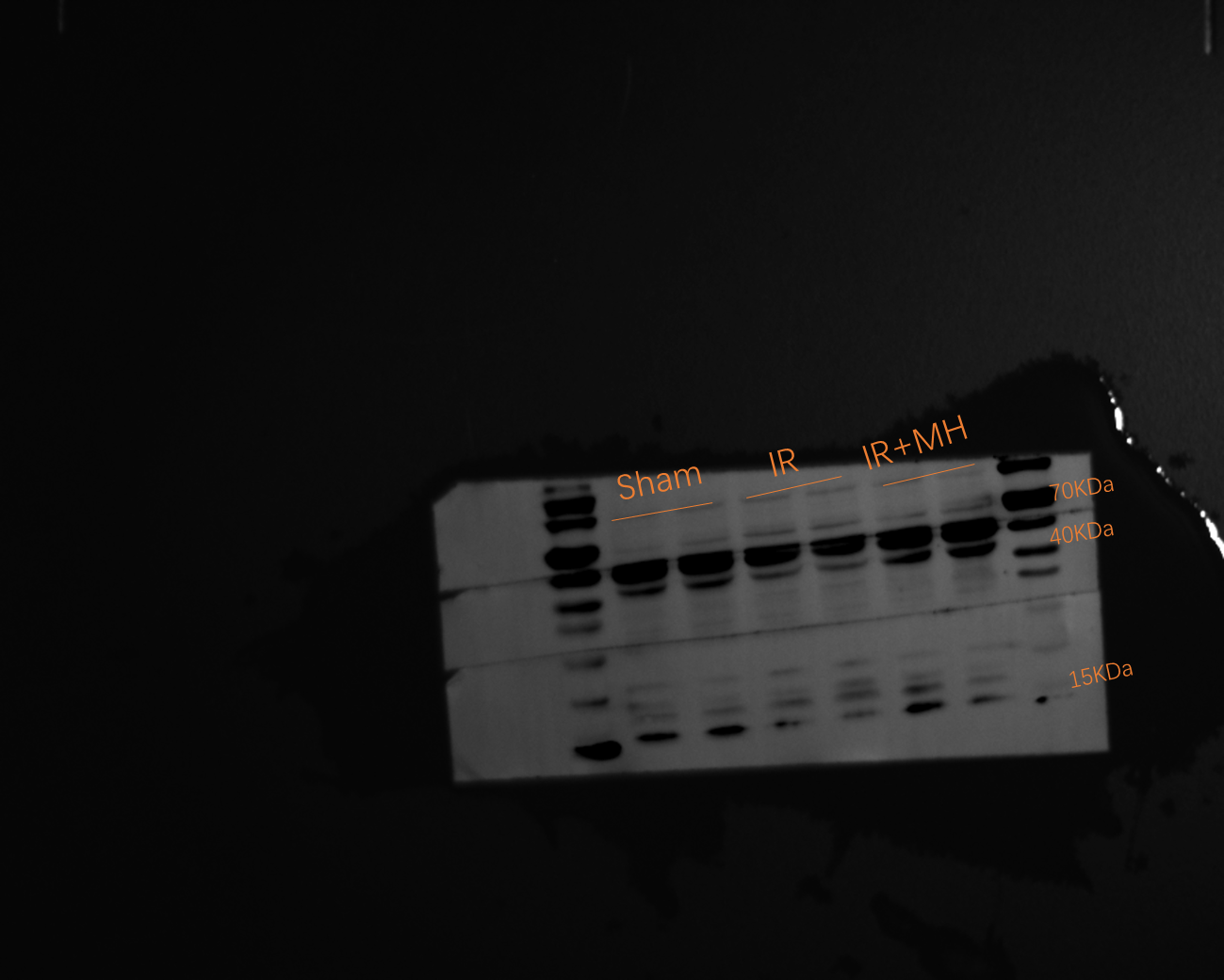

Supplement: Supplementary file 9 — Supplementary file9 (TIF 639 kb) [file 10571_2024_1483_MOESM9_ESM.tif]
